# Secondhand smoke prevention through the perceptions of pregnant women with smoking family members: a Thailand study

**DOI:** 10.1080/17482631.2024.2326109

**Published:** 2024-03-18

**Authors:** Sunisa Chansaeng, Waraporn Boonchieng, Warangkana Naksen

**Affiliations:** Faculty of Public Health, Chiang Mai University, Chiang Mai, Thailand

**Keywords:** Perceptions, pregnant women, prevention, second-hand smoke, smoking family members

## Abstract

**Purpose:**

Pregnant women with smoking family members are at risk of exposure to second-hand smoke, which leads to adverse pregnancy outcomes. Second-hand smoke prevention is thus important but remains less understood based on pregnant women’s perceptions. This study aimed to describe the perceptions of pregnant women on second-hand smoke prevention.

**Methods:**

This study employed a qualitative descriptive approach. Data collection was performed between July and August 2023 through in-depth interviews with 17 pregnant women purposively selected from a province in central Thailand. The verbatim transcribed data were analysed using thematic analysis.

**Results:**

Five themes emerged: unclear understanding of second-hand smoke; influences shaping perceptions related to second-hand smoke; attempt to prevent second-hand smoke exposure; barriers to prevention of second-hand smoke exposure; and needs related to prevention of second-hand smoke exposure.

**Conclusion:**

The findings provide insights into second-hand smoke prevention from the perception of pregnant women with smoking family members. Healthcare professionals need to design interventions tailored to pregnant women’s needs and involve smoking family members. It is necessary to develop and incorporate clinical guidelines into standard prenatal care to support healthcare personnel in identifying, assessing, educating, and mitigating the issue of second-hand smoking exposure.

## Background

Currently, 22% of adults worldwide who are 15 years of age or older consume tobacco (World Health Organization [WHO], 2023b), of whom 80% are living in low- and middle-income nations (WHO, 2021). In Thailand, it is estimated that each person smokes 11 cigarettes per day (National Statistical Office, 2023). As a result, a growing number of non-smokers have a greater likelihood to be exposed to passive tobacco smoke. Each year, tobacco use results in the deaths of over 8 million individuals worldwide, including roughly 1.3 million non-smokers who are exposed to second-hand smoke (WHO, 2023b). Pregnant women, in particular, are among persons with large exposure to second-hand smoke and their exposure is caused primarily by smoking family members. In Thailand, 11.7% of pregnant women reported second-hand smoke exposure, with 24.8% of them being exposed to second-hand smoke at home every day and 57.4% reporting having one smoking family members (Sonthon & Sonthon, 2021).

Second-hand smoke refers to a combination of smoke released by smokers and smoke produced when tobacco products burn (Sobh et al., 2021) and can harm persons exposed to it as much as or even more than the smokers themselves (WHO, 2023a). Second-hand smoke contains over 7,000 chemicals, including hundreds of harmful compounds and about 70 carcinogens (Centers for Disease Control and Prevention [CDC], 2022). Exposure to teratogenic agents in second-hand smoke during pregnancy can cause birth defects (Cunningham et al., 2022). Exposure to second-hand smoke increases the risk of preterm birth and foetal growth restriction (Ye et al., 2023), preterm birth (Wang et al., 2022), and low birth weight (Sonthon & Sonthon, 2021). The long-term impacts include asthma in children, problems with lung function, respiratory illnesses, and hypertension (Ye et al., 2023). Thus, prevention of second-hand smoke exposure is essential, particularly before 16 gestational weeks where major structural defects can occur (National Library of Medicine, 2021).

Extensive efforts have been initiated to keep non-smokers, including pregnant women, from being exposed to second-hand smoke. The WHO’s recommendations place a strong emphasis on the value of smoke-free homes and encourage pregnant women’s spouses and other family members to cut back on tobacco use (WHO, 2013). In the Thai context, the Family Development Promotion and Protection Act B.E. 2562 is enforced to avert domestic violence that includes any act a family member takes against another family member with intent to bring about, or which is probable to result in, harmful consequences to the family member’s life, body, mind, health, freedom, or reputation; or to force, or unjustly influence, a family member to engage in, abstain from, or comply with any action that is unlawful (The Secretariat of the Senate, 2020). Thus, smoking at home may fall under this criteria given that second- and even third-hand smoke has been shown to have a negative impact on family members’ health. Anyone who believes they are impacted by household smoking may report the matter to the relevant authorities, and inspectors will be dispatched to look into the matter and file charges against the smokers. However, this act does not specifically forbid smoking in homes and it does not mention the word “smoking”. Penalty against the smokers may be established through judicial interpretation. Moreover, the current health education for pregnant women in Thailand focuses on appropriate self-care behaviours during pregnancy and postpartum period where pregnant women are asked only about their history of smoking family members (Department of Health, Ministry of Public Health, 2022). Nevertheless, there are no actual guidelines or formal health education specific to the prevention of second-hand smoke for pregnant women.

Pregnant women prevent themselves and their foetus from second-hand smoke exposure by walking away, refusing to be in smoke-filled situation, not allowing people to smoke in their presence, and avoiding going to places where people regularly smoke (Yavagal et al., 2021), as well as asking smokers to stop smoking (D. A. Ayuningtyas, Tuinman, et al., 2021). However, their preventive behaviours of second-hand smoke exposure remain suboptimal, especially when the smokers are family members (Pookpan et al., 2021; Sonthon & Sonthon, 2019). Despite several quantitative studies on the practices of pregnant women’s second-hand smoke prevention in Thailand and other countries (Bayrami et al., 2021; Jantarasiew et al., 2021; Pookpan et al., 2021; Sonthon & Sonthon, 2019), an understanding of how pregnant women perceive second-hand smoke and prevention of second-hand smoke from family members is limited. This indicates the need to conduct a qualitative exploration of this phenomenon based on pregnant women’s perspectives, which will broaden an understanding of their views and practices of second-hand smoke prevention at home. This study sought to describe the perceptions of pregnant women with smoking family members on second-hand smoke prevention.

## Method

### Study design

We used a qualitative descriptive methodology to provide a thorough, relatable summary of the individual experiences with certain occurrences (Lambert & Lambert, 2012). In order to extract and record testimony of people’s distinct experiences with practices, naturalistic inquiry served as the foundation for the data collection procedure (Sandelowski, 2000). This research employed descriptive inquiry to improve applicability across practitioners, and instead of presenting the findings in abstractive, conceptual, or philosophical terms, the authors used everyday language (Sandelowski, 2000).

### Participants

The participants were pregnant women who visited an antenatal clinic of a sub-district health promoting hospital in central Thailand (Table I). The research project was advertised by distributing flyers at the sub-district health promoting hospital. Pregnant women who were interested in participating contacted the researcher via the telephone number given in the flyers. Then, they purposively selected based on the inclusion criteria: age 18 and over; gestational age of less than 16 weeks; non-smoker; no complications or comorbidity during pregnancy; and living with a smoking family member. The research aims, procedures, confidentiality, risks, advantages, contribution, and ability to refuse or withdraw from the study were all explained to the eligible participants. Data saturation in this study was reached with 17 participants when no further information could be gleaned from the interviews.

**Table I.** Description of the characteristics of 17 participants.

Participant	Age (years)	Education	Occupation	Monthly personal income Thai baht (USD)	Number of family smokers	Family smoker
1	20	Elementary	Merchant	8,000 (227.60)	1	Husband
2	18	Elementary	Unmployed	–	1	Husband
3	19	Elementary	Unmployed	–	1	Husband
4	27	Junior high school	Unmployed	–	2	Husband and father
5	25	Vocational certificate	Merchant	15,000 (426.74)	1	Husband
6	18	Senior high school	Duck farmer	13,000 (369.84)	1	Husband
7	27	Senior high school	General labourer	14,000 (398.29)	2	Husband and father
8	30	Junior high school	General labourer	10,000` (284.50)	1	Husband
9	17	Senior high school	Student	–	2	Husband and father
10	26	Elementary	General labourer	30,000 (853.49)	1	Husband
11	18	Junior high school	Unmployed	–	1	Husband
12	28	Bachelor’s degree	General labourer	18,000 (512.09)	1	Husband
13	24	Bachelor’s degree	General labourer	20,000 (568.99)	1	Husband
14	33	High vocational certificate	General labourer	15,000 (426.74)	1	Husband
15	29	Junior high school	Merchant	25,000 (711.24)	2	Husband and father
16	31	Bachelor’s degree	General labourer	30,000 (853.49)	1	Husband
17	25	Senior high school	Farmer	15,000 (426.74)	1	Husband

### Data collection

Data were collected between July and August 2023. The reporting of this study complied with the Consolidated Criteria for Reporting Qualitative Research (COREQ) (Tong et al., 2007). Before the study started, there were no prior relationships between the participants and the researcher. As a result, rapport was established and maintained until the end of the interview. The first author, a female doctoral candidate, conducted the interviews in the participant’s house or in the counselling room of the antenatal clinic. Each participant was interviewed in two to three sessions, lasting between 40–60 minutes per session. The interviews were done in a discreet and convenient location. Open-ended questions from the semi-structured interview guide were used in the interviews, including *“Do you know or have ever heard of secondhand smoke? What is it?” “What do you think about cigarettes and secondhand smoke? Is there any danger or effect?” “What are the ways to protect yourself from cigarette smoke in the home?” “How does your antenatal clinic organize health promotion activities for pregnant women and their husband?” “How should health education be taught to pregnant women on protecting themselves from secondhand smoke?”* The researcher used probing approaches by posing questions on the crucial topics in order to obtain sufficient data, a depth and breadth of interpretations, and a comprehensive description of the participants’ perspectives. Audio recordings of the interviews were made with permission.

### Data analysis

The collection and analysis of the data were done simultaneously. Thematic analysis was used to manually evaluate the data (Sandelowski, 2000). We carried out the subsequent steps: 1) listen to the participants’ stories repeatedly; 2) transcribe the interviews verbatim in the Thai language; 3) read and reread the transcriptions several times for comprehensive understanding; 4) code the data; 5) categorize the codes into sub-themes; 6) identify related sub-themes within themes; and 7) examine and enhance the themes and sub-themes in light of the literature and research questions. Finally, an English translation of the findings was made.

### Trustworthiness/Rigor

Credibility, transferability, dependability, and confirmability were employ to assure trustworthiness (Lincoln & Guba, 1985). Member checking with three participants to go over potential themes was used to determine credibility. Regarding the preliminary findings, none of them offered opposing viewpoints. Two peer debriefings with qualitative research specialists were held to confirm the procedure for gathering and analysing data. By providing comprehensive details on participants and study contexts, transferability was attained. In order to ensure dependability, five pregnant women with similar characteristics to the study participants were used for pilot interviews to test the interview guide. Based on their responses, the interview guide was modified. Data analysis, conclusions, and interpretation were discussed with the advisory committee. In order to minimize bias and guarantee correct data interpretation, confirmability was achieved by keeping a reflexive journal and field notes for every interview both before and after data collection, as well as throughout data processing.

## Ethical considerations

This study was approved by the research ethics committee of a university (Document No. ET034/2023). The research aims, procedures, confidentiality, risks, advantages, contribution, and the ability to refuse or withdraw from the study were all explained to the eligible women. Written consent was given by each participant. Code numbers were used to guarantee participant anonymity. During the interview, participants were assessed for physical and pshychological discomfort. In this study, none of the participants showed signs of discomfort that required referral for further professional assistance. No monetary incentives were given to the participants.

### Theme 1: unclear understanding of secondhand smoke

The majority of the participants mentioned an unclear understanding of second-hand smoke in terms of non-recognition of second-hand smoke, misperception of second-hand smoke, and unawareness of harms from second-hand smoke.

#### Non-recognition of secondhand smoke

When asked about their knowledge of second-hand smoke, around a quarter of the participants did not know what second-hand smoke was, as they had never heard of it before.

What is secondhand smoke? Is it dangerous? I’m not sure. (Participant 2)

I’ve never heard of secondhand smoke before. Is it dangerous? (Participant 11)

I don’t know about secondhand smoke. What is it? (Participant 4)

Moreover, some participants did not clearly know about the harmful substances in second-hand smoke. Some of them could identify certain common substances, but did not know how the harmful substances might affect the unborn baby.

What substances are in secondhand smoke? All I know is there’s nicotine but is nicotine released from secondhand smoke? (Participant 2)

What are the substances in secondhand smoke? How can they harm an unborn baby? (Participant 6)

#### Misperception of secondhand smoke

A quarter of the participants understood that second-hand smoke was similar to other kinds of smoke in their daily life, such as smoke from the car’s exhaust, smoke from burning, or particulate matter of 2.5 microns or less in diameter (PM2.5).

What does secondhand smoke look like? Is it like the smoke that’s coming out of the car’s exhaust? (Participant 8)

Secondhand smoke is like smoke from burning. When I smell it, I can’t breathe. (Participant 3)

I think secondhand smoke is like other kinds of smoke in general … like PM2.5. (Participant 7)

#### Unawareness of harms from secondhand smoke

Roughly half of the participants thought that the harms of second-hand smoke were not as serious as those faced by the smokers. They believed that the smoke did not directly reach their lungs, so the health consequences would not be severe.

The harms from secondhand smoke may be different from the harms for the smokers. I don’t smoke so it may be less harmful. I think pregnant women who smoke are more affected than those who don’t smoke or those who are exposed to secondhand smoke from their husband. (Participant 12)

I don’t think secondhand smoke is a problem to me because I’m not the one who smokes. The harms wouldn’t be too serious. It should be fine. I wouldn’t get affected like people who smoke. The smokers inhale the smoke directly into their lungs, but I can swing my hands to push the smoke away so the smoke doesn’t get into my lungs. (Participant 4)

A participant believed that the unborn baby would not be affected by second-hand smoke because she associated the harms to the smell, which could not reach the baby.

Secondhand smoke may not affect the baby because the baby is in the womb. How can the baby smell anything? They baby may be affect by what I eat, but the smell can’t get to the baby. (Participant 9)

Two participants whose husbands’ occupation required travelling and frequent overnight stays also thought they were safe from the harms of second-hand smoke because their husband smoked when they were away.

My boyfriend^1^ smokes but we rarely see each other. He likes to smoke while working but doesn’t smoke when he’s with me. He smokes in front of the house. When he’s done smoking, he walks in. So it would not have any effect on me. (Participant 14; husband works in logistics)

I don’t think my boyfriend’s smoking is a problem for my pregnancy because we don’t see each other much. He smokes when he goes to work on a farm. (Participant 17; husband works in farming)

### Theme 2: influences shaping perceptions related to secondhand smoke

Participants’ perceptions of second-hand smoke and its harms were shaped by various influencial sources, including their own personal experience, laypeople, healthcare providers, and mass media.

#### Personal experience

Some participants based their perceptions of the harms from second-hand smoke on the health effects on their previous pregnancy, which caused them to fear the adverse consequences of second-hand smoke.

When I was pregnant with my first child, my ex-boyfriend and I smoked because at that time I was a teenager and didn’t think anything of it. But my first child is not healthy at all. He gets sick a lot and has asthma. So we quit smoking. When I found out I was pregnant this time, I’m afraid my baby would have health problems like my first child. (Participant 13)

My boyfriend doesn’t smoke near me now because he’s afraid the baby would end up with health problems like our first child. (Participant 9)

However, some personal experience led to confidence to continue exposure to second-hand smoke and neglect the harms.

My father has smoked since I was born and still smokes until now. I don’t see anything wrong with my health. So I don’t care about cigarette smoke. (Participant 10)

#### Laypeople

About a quarter of the participants revealed that their friends, relatives, and people they knew were important in shaping how they viewed second-hand smoke.

One of my friends had miscarriage. She said her boyfriend usually blew cigarette smoke to her belly. He said the baby liked it. Then, the baby stopped moving. I think the miscarriage could have been caused by cigarette smoke. (Participant 16)

There is someone close to me … She’s my relative. Her boyfriend smoke a pack of cigarettes daily while she was pregnant. Her baby was born with low birth weight and needed to be in the NICU [neonatal intensive care unit] on a ventilator for months. Seemed like the baby had problems with his lungs. (Participant 12)

#### Healthcare providers

As shared by a few participants, their perceptions of second-hand smoke were sometimes derived from healthcare providers’ history taking about second-hand smoke and advice on its avoidance. This allowed them to learn that second-hand smoke was harmful to an unborn baby.

I think cigarette smoke affects pregnancy because a nurse asked me about the history of smoking and gave me advice about my boyfriend’s smoking that he should not smoke near me. Otherwise, the baby’ll be in danger and may be born with disability, or have asthma or allergies. (Participant 13)

The nurses the hospital where I got antenatal care for my first child once said that cigarette smoke was dangerous for unborn babies. (Participant 9)

#### Mass media

Information about second-hand smoke and its harms was also obtained from the mass media such as television commercials and social media video clips.

I saw on TV [television] commercials that the harms of being exposed to cigarette smoke from others were equally the same as those faced by the smokers themselves. (Participant 3)

I think secondhand smoke is harmful. From the clips I’ve seen, it can cause miscarriage. (Participant 7)

I’ve heard from social media that secondhand smoke can cause miscarriage if exposed in the first months of pregnancy, and can cause the baby to die in their sleep. (Participant 10)

### Theme 3: attempt to prevent secondhand smoke exposure

Almost all of the participants tried to prevent themselves and their unborn baby from exposure to second-hand smoke through two main methods, including avoidance and taking action to intervene with smoking.

#### Avoidance

Although most of the participants wanted to ask the family smokers to stop smoking, they did not do it because they feared that it would bring about family conflicts. Therefore, in order to maintain family relationship, they chose to avoid coming close to the smoking family members by walking away.

I chose to walk away when my husband smokes. I don’t want to tell him not to smoke. I don’t want it to turn into an argument. I do whatever makes him happy. I don’t want to cause tension. (Participant 10)

I walk away from him. Don’t want to tell him not to smoke because we’ll end up fighting. (Participant 5)

#### Taking action to intervene with smoking

Sometimes, around a quarter of the participants took action by asking smokers to reduce smoking, smoke far away from them, or wash themselves before coming in the house.

I can smell the cigarette so I tell him to reduce smoking. I also tell him to leave the room, not to get near me, and take a shower. I don’t like cigarette smell. It’s very strong. (Participant 5)

When he wants to smoke, I tell him to smoke somewhere else. He does what I ask for. He would smoke in front of the house. On some days, he doesn’t even smoke at home. (Participant 12)

### Theme 4: barriers to prevention of secondhand smoke exposure

In their endeavour to prevent second-hand smoke exposure, most of the participants mentioned facing various barriers, including having no time to seek information, lack of health education coverage on second-hand smoke, powerlessness, smoker’s disbelief of second-hand smoke consequences, limited space, and social triggers.

#### No time to seek information

Some of the participants disclosed that they had to work hard to earn a living, which caused them to be busy and have no time to search for information about second-hand smoke.

I don’t have time to learn about secondhand smoke or cigarettes. I have to work and I come home very tired. (Participant 10)

Nurses told me that secondhand smoke was harmful to the baby, but I had to work. I was busy so I didn’t look for more information. (Participant 9)

If I were a stay-at-home mom, I would have more time to search for information about what’s best for my baby. But I have to work every day. I don’t even have time to use my phone. (Participant 15)

#### Lack of health education coverage on secondhand smoke

The topics of health education mainly focused on abused substances and condom use rather than second-hand smoke and its prevention.

The health education doesn’t emphasize cigarette smoke or prevention of its exposure. They focus only on abused substances and condom use. (Participant 11)

Does cigarette smoke have anything to do with pregnancy? They don’t mention this in parent education class. (Participant 15)

Moreover, a participant revealed that she did not know whether the non-smokers were protected by laws, implying that legal protection and rights of non-smokers are not well taught.

Is there any legal protection for those who don’t smoke? If there is, it’d be great because my boyfriend respects the law. He’s afraid of the police. (Participant 14)

#### Powerlessness

A significant proportion of the participants felt powerless in making their requests fulfilled when it came to asking a family smoker to stop smoking.

My boyfriend’s smoking is the main problem to my pregnancy. It’s easy for me to ask others not to smoke in the home, but when it’s my boyfriend, I can’t get him to listen to me. He never believes in anything I say. He said I’m not well educated so I know little. (Participant 7)

For my husband … when the doctor tells him not to smoke in the home or near me, he believes the doctors and does it. He believes others, not me. (Participant 10)

#### Smoker’s disbelief of secondhand smoke consequences

Despite participants’ effort to keep their unborn baby safe from second-hand smoke, their smoking family members disagreed and refused to cooperate. The smoking family members did not see the need to quit smoking because they did not believe in the consequences of second-hand smoke on the non-smokers. They believed that only the smokers themselves would be affected by cigarette smoke.

My husband thinks the smokers will get the consequences of cigarette smoke, not me or my unborn baby. He says it’s his lungs, not my lungs. (Participant 12)

My boyfriend says he’s the one who smokes, so the smoke will get into his lungs only. He says the one who is affected is the smoker. He says the smoke will blow away. It won’t get inhaled into my lungs. (Participant 15)

#### Limited space

Limited living space was another important impediment, causing some of the participants to be unable to completely avoid second-hand smoke exposure.

We live in a studio apartment with limited space, so my husband has to smoke in home. He doesn’t want to bother our neighbors with the smell. Our apartments are right next to each other. None of our neighbors smoke. (Participant 2)

We live in a rented studio apartment so we have limited space. My husband has to smoke indoors. (Participant 13)

#### Social trigger

Living with others who smoke triggered the smoking family member to continue smoking and made it even more difficult for many participants to intervene.

At home, many family members smoke. Besides my husband, my father also smokes. Now we all live together, so it’s like everyone smokes. I can’t forbid them. When they see each other, they hang out, drink, and smoke. (Participant 6)

All of my male relatives smoke, so I can’t forbid them. They always smoke when they see each other. I’m the one who gets scolded when I complain and try to ask them not to smoke. (Participant 15)

Additionally, visiting public places where people smoked or social gatherings with other smoking people tempted the family members to smoke.

There’re always people smoking everywhere we go, so my boyfriend can’t resist the temptation to smoke. (Participant 10)

When his colleagues visit and hang out at our home, they always drink and smoke, right in front of our home. They don’t care if I’m around. (Participant 13)

### Theme 5: needs related to prevention of secondhand smoke exposure

To facilitate prevention of second-hand smoke, most of the participants mentioned a variety of needs for health education about second-hand smoke and prevention, inclusion of smoking family members, and peer support group.

#### Health education about secondhand smoke and prevention

Approximately half of the participants needed to learn more about the potential effects of second-hand smoke on pregnancy outcomes, as one said,*“I really want to know what consequences of secondhand smoke are on the baby.”* (Participant 5) In particular, they voiced the need for innovative media for health education on various social media and online platforms in forms of video clips and picture-based to facilitate better understanding.

TikTok. I like to watch video clips about pregnancy and harmful drugs that could affect the baby. I like to read comic books. I prefer to look at pictures because they make me understand better. (Participant 6)

I want health education to include pictures and disseminated via Line application so I can access them whenever I want. (Participant 9)

Some of the participants emphasized that health education should be brief with summary of the main points, and should be based on non-medical terms for laypeople’s understanding.

I prefer YouTube, but the clips should be short, under 10 minutes. It’d be boring with too much content. (Participant 1)

I want the media to be easy to understand. I’m not well educated so I don’t understand difficult terms. (Participant 10)

#### Inclusion of smoking family members

Roughly half of the participants also expressed the need to include their smoking family members in health education about second-hand smoke in order to improve the family smokers’ understanding of harms of second-hand smoke and proper practices for preventing exposure.

I want doctors to teach and explain about the harms of cigarette smoke. I want my boyfriend to come as well so he’ll be able to do it right. I want him to join every session of health education. (Participant 16)

I want my husband to attend health education so he’ll know that he shouldn’t smoke near pregnant women. He’ll be able to ask questions if he doesn’t understand anything. If secondhand smoke is harmful, then I think my husband should be involved in the discussions with health providers. (Participant 17)

Moreover, inclusion of family members in health education was believed to lead to success in smoking cessation.

I believe it’ll be beneficial if my boyfriend comes to health education on smoking. He wanted to quit smoking, but he couldn’t. (Participant 6)

#### Peer support group

Around one-third of the participants needed a peer support group where pregnant women with smoking family members could share their experiences, which could be via social media and online platforms.

I want to have a group where we can share our experiences or health information via Line application, so we can learn more about the harms of secondhand smoke. (Participant 11)

I want to listen to others’ experiences. It’s not boring. We can also join a group chat on Line application, so we can ask each other questions whenever we want. (Participant 8)

## Discussion

The findings disclosed the perceptions of pregnant women on second-hand smoke prevention that emerged in four themes (Table II).

**Table II.** Themes, sub-themes, and codes.

Theme	Subthemes	Codes
Theme 1: Unclear understanding of second-hand smoke	1.1 Non-recognition of second-hand smoke	Not know what second-hand smoke was Not sure Never heard of Not know the substances in second-hand smoke
	1.2 Misperception of second-hand smoke	Like the smoke from the car’s exhaust Like smoke from burning Like PM2.5
	1.3 Unawareness of harms from second-hand smoke	Less harmful Not serious Can’t reach the baby Not living with the smoker
Theme 2: Influences shaping perceptions related to second-hand smoke	2.1 Personal experience	Previous pregnancy First child is not healthy. Afraid the baby would have health problems like the first child
	2.2 Laypeople	Friend’s boyfriend Relative’s boyfriend
	2.3 Healthcare providers	Taking history of smoking Health advice Antenatal care
	2.4 Mass media	Television commercials Social media clips
Theme 3: Attempt to prevent second-hand smoke exposure	3.1 Avoidance	Walk away Not want to cause conflict
	3.2 Taking action to intervene with smoking	Tell the smoker to reduce smoking Tell the smoker to leave the room Tell the smoker not to get near Tell the smoker to smoke elsewhere
Theme 4: Barriers to prevention of second-hand smoke exposure	4.1 No time to seek information	Not have time to learn about second-hand smoke Have to work Busy
	4.2 Lack of health education coverage on second-hand smoke	Not emphasize cigarette smoke or prevention Not mention in parent education class
	4.3 Powerlessness	Cannot get the smoker to listen Cannot get the smoker to believe in what they say
	4.4 Smoker’s disbelief of second-hand smoke consequences	Smokers get the consequences of cigarette smoke. The smoke will get into the smoker’s lungs only.
	4.5 Limited space	Live in a studio apartment Little space
	4.6 Social trigger	Everyone smokes. All male relatives smoke. Hang out, drink, and smoke People smoking everywhere
Theme 5: Needs related to prevention of second-hand smoke exposure	5.1 Health education about second-hand smoke and prevention	Picture-based TikTok YouTube Line application
	5.2 Inclusion of smoking family members	Want husband to come Want husband to attend health education Husband should be involved.
	5.3 Peer support group	A group to share experience A group to share information A group chat

### Unclear understanding of secondhand smoke

We found that the participants still had an unclear understanding of second-hand smoke in terms of what second-hand smoke was, the substances in it, or the consequenes on unborn babies. Likewise, pregnant women in India (Yavagal et al., 2021) and Vietnam (Vu et al., 2020) lacked knowledge and awareness of health issues on infants caused by second-hand smoke. Pregnant women did not consider the risks associated with second-hand smoke exposure and were therefore unconcerned about prenatal exposure for themselves and their developing foetus (Artzi-Medvedik et al., 2022). Our participants did not find their partners’ smoking as a concerning issue of them as they believed that health consequences of second-hand smoke would not be as serious as those for the active smokers and their partners did not smoke near them, which was in line with other studies (D. A. Ayuningtyas, M. A. Tuinman, et al., 2021; Khanal et al., 2018). In fact, it is important that a no tobacco smoke environment at home be recommended for pregnant women and completely eliminating smoking is the only way to fully protect people who do not smoke from second-hand smoke exposure (Centers for Disease Control and Prevention, 2022). Although some of the participants mentioned receiving information on second-hand smoke during antenatal care, such information was simply for history taking and included a warning that second-hand smoke was harmful, but not in detail. The inadequate understanding of second-hand smoke might be attributable to the lack of health education coverage on second-hand smoke in Thailand, which only asks pregnant women about their history of family smoking but does not include information on what second-hand smoke is, the harmful substances in second-hand smoke, or the consequences of second-hand smoke on pregnancy (Department of Health, Ministry of Public Health, 2022). This lack of knowledge emphasizes the necessity of further initiatives to raise this population’s understanding of the consequences of second-hand smoke because knowledge and awareness are significant factors influencing the behaviours of pregnant women in preventing exposure of second-hand smoke at home (Oktalia, 2023).

### Influences shaping perceptions related to secondhand smoke

The participants’ perception related to second-hand smoke was shaped by various sources. Participants drew upon their own experiences in previous pregnancies to determine the health effects of second-hand smoke on the unborn baby. Other sources, such as laypeople, healthcare providers, and mass media, also played an important role in informing about second-hand smoke. Similarly, Thai pregnant women based their perspectives of harmful substances on the outcomes of their previous pregnancies, the accounts of harms occurring to their friends’ children, and doctors’ warnings (Tantanokit et al., 2023). Nevertheless, it is noteworthy that some personal experinces where no obvious harms were present with their own or others’ former pregnancy despite exposure to second-hand smoke might led to underestimation of harms (Artzi-Medvedik et al., 2022). Although the significant persons in the pregnant women’s context can influence their perceptions and potentially help to increase their awareness of the harms, there is undeniably a necessity to increase pregnant women’s capability to judge the reliability of information from various sources, particularly those that are not from the professionals. As one of the trusted sources of information, healthcare providers can take advantage by enquiring about second-hand smoke exposure and advising pregnant women regarding consequences of second-hand smoke exposure in terms of adverse birth outcomes, and the need to create non-smoking home environments.

### Attempt to prevent secondhand smoke exposure

We found that the participants attempted to prevent second-hand smoke exposure through avoidance and taking action to intervene with smoking. Our findings were consistent with other studies that common strategies to prevent second-hand smoke exposure among pregnant women were involved avoidance by withdrawing from smoking situations (Artzi-Medvedik et al., 2022; Mazloomy Mahmoodabad et al., 2019) and proactive actions by setting a non-smoking rule at home (Pookpan et al., 2021). Interestingly, our participants chose to avoid the smokers by walking away because they needed to maintain good family relationship and they feared that confronting the smokers would potentially lead to an argument. This practice might be attributable to the context of Asian culture. Congruently, the fear of jeopardizing relationships and the fact that a wife had to consider her husband’s feelings were mentioned reasons not to intervene with the partner’s smoking (D. A. Ayuningtyas, Tuinman, et al., 2021). However, men can safeguard women’s health during pregnancy by participating in educational initiatives and counselling. Men’s involvement in the health program designed for pregnant women might influence social and behavioural changes in them as well as motivate them to assume greater responsibility for the health of their wives and children (Bayrami et al., 2022).

### Barriers to prevention of secondhand smoke exposure

Participants stated that they encountered a number of obstacles in their efforts to avoid being around second-hand smoke. As most of the participants were employed and had to work hard to support their family, they had no time to seek information about second-hand smoke. They also mentioned limited living space as another barrier, causing them unable to distance themselves from their partner’s smoking. These barriers suggest that socioeconomic situation is an important factor of second-hand smoke particularly in low- and middle-income countries (Zhou et al., 2022). As participants received information about second-hand smoke from health care professions and social media, they might not feel the need to proactively search for more information. Moreover, the available health education did not cover second-hand smoke and protection of non-smokers by laws, which limited participants’ action to prevent second-hand smoke exposure. Education is one of the fundamental forces that can encourage positive behaviour and shield people from dangerous exposure. Interestingly, some of the participants reported obtaining information related to second-hand smoke from their healthcare providers. This suggests that some healthcare providers may be better than others at delivering this information. A study involving 367 health professionals revealed that only half of health professionals had good knowledge of second-hand smoke and effective counselling practices for second-hand smoke, which was predominantly caused by inadequate training (Hassanein et al., 2022). Therefore, it is vital to train healthcare providers how to effectively deliver health information. Powerlessness and smoker’s disbelief of second-hand smoke consequences were also identified as impeding women’s success in prevention of second-hand smoke exposure. These barriers are not surprising in Asian context where women are expected to be obedient to their spouses, less likely to change the smoking behaviour of their partners or male family members, and report frequent exposure to second-hand smoke (D. A. Ayuningtyas, M. A. Tuinman, et al., 2021; Zhou et al., 2022). Thus, empowering pregnant women to be confident in taking action with regard to smoking at home would be a beneficial step to reduce exposure. As our findings revealed that husbands tended to believed what the doctors recommended, this should be an opportunity to educate husbands and raise their awareness of the harms of second-hand smoke while promoting pregnant women’s roles in making their voice heard. An education intervention where pregnant women and their husband worked together as co-partners in their endeavour to maintain a smoke-free home was effective in enabling pregnant women’s success in increasing their husband’s awareness of second-hand smoke (Karimiankakolaki et al., 2023). We also found that social triggers led to difficulty to manage second-hand smoke exposure. Research consistently showed that smoking habits were influenced by smoking friends or co-workers (D. A. Ayuningtyas, M. A. Tuinman, et al., 2021). Family, the society, and cultural backgrounds add complexities to tackling the issue of second-hand smoke, particularly in patriarchal settings.

### Needs related to prevention of secondhand smoke exposure

In preventing second-hand smoke, the participants emphasized several needs. They needed health education about second-hand smoke and prevention, which should be brief and easy-to-understand, and employ innovative media, such as online or social media platforms. This need reflects that the health interventions should be tailored to the literacy level of pregnant women and their modern lifestyle. Consistently, pregnant women were eager to learn about and receive information about reducing their exposure and needed individualized, accessible, and practical health education in this issue (Artzi-Medvedik et al., 2022). Electronic technology proved useful in increasing access, encouraging participation, and distributing information to all expectant mothers (Hamadneh & Hamadneh, 2023). Our participants also expressed the need for the inclusion of smoking family members in health education, suggesting that the success in preventing second-hand smoke exposure does not only depend on the pregnant women alone but also on the understanding and willingness of the smoking family members, especially their partners. Expectant fathers have an ideal opportunity to encourage healthy behaviours since they may be more receptive to behavioural interventions and health-related information (Xia et al., 2021). Therefore, interventions that educate pregnant women’s partners to be aware of the harms of second-hand smoke may help to enhance men’s concerns and motivation to quit smoking. The last need was to have a peer support group where pregnant women can share their experiences regarding second-hand smoke. This finding echoes another study that women found that other pregnant women were a useful network of support who were aware of and sympathized with what they were going through (Weiland et al., 2022). An opportunity involving witnessing or hearing about other women’ experiences of fighting against second-hand smoke might encourage pregnant women to continue in their endeavour.

### Limitations

Our study had some limitations. The transferability of qualitative findings is limited to similar social and cultural contexts, and may not represent all pregnant women population. This study may be strengthened by a larger sample of participants with varying educational, familial, and social backgrounds.

## Conclusions

The findings offers second-hand smoke prevention from the perspectives of pregnant women with smoking family members. Healthcare providers need to create interventions tailored to the diverse needs and contexts of pregnant women with engagement of the smoking family members to enhance their awareness of second-hand smoke and empower pregnant women to prevail in their endeavour to achieve smoke-free homes. Clinical guidelines need to be created and integrated into routine prenatal care to help healthcare providers assess, recognize, educate, and mitigate the effects of second-hand smoke exposure.

## Supplementary Material

Semistructured interview guide_SUPPLEMENT.docx

## Note

1. * In Thai culture, women sometimes refer to their husband as “boyfriend.”
