# Supplementary material for: Secondhand smoke prevention through the perceptions of pregnant women with smoking family members: a Thailand study
Source: Int J Qual Stud Health Well-being. 2024 Mar 18;19(1):2326109. doi: 10.1080/17482631.2024.2326109 (PMC10949832; doi:10.1080/17482631.2024.2326109)
Supplement: Semistructured interview guide_SUPPLEMENT.docx [file ZQHW_A_2326109_SM1691.docx]

**Semi-structured interview guide for pregnant women**

Date......................................

**About the participant:**

1. Age ................years

2. Education.....................................................

3. Occupation....................................................

4. Monthly income……………………baht

5. Number of smoking family members ............................ Please identify who ................................

**Interview questions**

1. What are health promotion activities offered by the antenatal clinic for pregnant women and their husbands?

2. What is secondhand smoke in your opinion?

3. In your opinion, what are the harms of cigarette smoke or secondhand smoke?

4. What are the effects of secondhand smoke from your family member(s) on your pregnancy? How do you prevent yourself from exposure to secondhand smoke?

5. What should health education for prevention of secondhand smoke exposure include? Who should be involved in the health education?

6. What should health education materials for prevention of secondhand smoke exposure be like?
